# A Novel, Combined Student and Preceptor Professional Development Session for Optimizing Feedback: Protocol for a Multimethod, Multisite, and Multiyear Intervention

**DOI:** 10.2196/32829

**Published:** 2022-06-13

**Authors:** Brenton Button, Clare Cook, James Goertzen, Erin Cameron

**Affiliations:** 1 Human Sciences Division Northern Ontario School of Medicine University Thunder Bay, ON Canada; 2 Medical Education Research Lab in the North Northern Ontario School of Medicine University Thunder Bay, ON Canada; 3 Faculty of Education University of Winnipeg Winnipeg, MB Canada; 4 Continuing Education and Professional Development Northern Ontario School of Medicine University Thunder Bay, ON Canada; 5 Centre for Social Accountability Northern Ontario School of Medicine University Thunder Bay, ON Canada

**Keywords:** feedback, professional development, undergraduate, medical education, intervention, preceptors, students, medical students, longitudinal integrated clerkship

## Abstract

**Background:**

Providing feedback to medical learners is a critical educational activity. Despite the recognition of its importance, most research has focused on training preceptors to give feedback, which neglects the role of learners in receiving feedback. Delivering a combined professional development session for both preceptors and students may facilitate more effective feedback communication and improve both the quality and quantity of feedback.

**Objective:**

The objective of our research project is to examine the impact of a relational feedback intervention on both preceptors and students during a longitudinal integrated clerkship.

**Methods:**

Students and preceptors will attend a 2.5-hour combined professional development session, wherein they will be provided with educational tools for giving and receiving feedback within a coaching relationship and practice feedback giving and receiving skills together. Before the combined professional development session, students will be asked to participate in a 1-hour preparation session that will provide an orientation on their role in receiving feedback and their participation in the combined professional development session. Students and preceptors will be asked to complete a precombined professional development session survey and an immediate postcombined professional development session survey. Preceptors will be asked to complete a follow-up assessment survey, and students will be asked to participate in a follow-up, student-only focus group. Anonymized clinical faculty teaching evaluations and longitudinal integrated clerkship program evaluations will also be used to assess the impact of the intervention.

**Results:**

As of March 1, 2022, a total of 66 preceptors and 29 students have completed the baseline and follow-up measures. Data collection is expected to conclude in December 2023.

**Conclusions:**

Our study is designed to contribute to the literature on the feedback process between preceptors and students within a clinical setting. Including both the preceptors and the students in the same session will improve on the work that has already been conducted in this area, as the students and preceptors can further develop their relationships and coconstruct feedback conversations. We will use social learning theory to interpret the results of our study, which will help us explain the results and potentially make the work generalizable to other fields.

**International Registered Report Identifier (IRRID):**

DERR1-10.2196/32829

## Introduction

### Background

Key components for the training of physicians are precepting and providing supervision within clinical settings. Clerkships are full learning immersion experiences wherein, under the supervision of a preceptor, students have an opportunity to learn by performing different tasks, ranging from taking patient histories to collaborating on diagnosis and treatment while assisting with the provision of patient care [1]. Clerkships are a highly impactful educational experience that can vary greatly among communities, hospitals, rotations, and preceptors. Research has suggested that the quality of a preceptor and their relationship with a student in a clerkship can significantly impact the overall placement and learning experience [2]. One critical component of the student-preceptor relationship is the quality of the feedback that a preceptor provides to a learner. In fact, it has been suggested that feedback is the foundation of effective clinical teaching, and providing feedback has been identified as a key physician competency (scholar role) within the CanMEDS framework [3,4].

Feedback occurs when a learner is offered insight into how they actually performed and the consequences of their actions [5]. Providing feedback can help learners maximize their performance at different stages of training, and it can assist learners in recognizing their strengths and areas for improvement and identifying actions that can be taken to improve performance. Since the early 1980s, feedback in medical education has been recognized as being important, and giving feedback is far from simple. Preceptors have concerns about giving negative feedback, and each student has their own comfort level and needs when receiving feedback [5]. An integrative review of the content of teacher-learner feedback found that preceptors were reluctant to give critical feedback, preceptors provided low-quality feedback, and feedback sessions were dominated by the preceptors [6]. These results indicate that providing feedback has remained a challenge over the last 3 decades.

One of the early approaches to giving feedback featured a unidirectional approach wherein a preceptor surrounds a piece of criticism with 2 pieces of praise—the so-called *feedback sandwich* [7]. In some instances, this approach resulted in dissatisfaction as the preceptor initiated the feedback process, leaving the receiver without enough quality feedback. Some workshops were created to alleviate this problem and help students elicit feedback from their preceptors. This approach had benefits but placed the onus of obtaining feedback on the receiver [8]. In response, an educational alliance framework for improving feedback effectiveness was proposed. An educational alliance framework posits that feedback needs to be changed from an “information download” to a bidirectional conversation in an authentic partnership that includes mutually agreed upon performances and standards, a coconstructed action plan, teamwork, and the purposeful use of a feedback discussion in practice [9]. Using an educational alliance framework has been suggested as an effective way to promote a feedback culture in *Medical Teacher’s* popular *Twelve Tips* section [10].

Research that uses an educational alliance to frame an intervention has had moderate success but suggests that one major barrier is the lack of interest from clinical teachers [11]. Another barrier is the challenge with establishing relationships [12].

One method that has been used and can work with an educational alliance framework is a coaching approach. Coaching involves observing a task and then using different actions, questioning tactics, or encouragement to improve performance [13]. Using a coaching approach might help with establishing stronger relationships by building mutual trust, promoting engagement with educational content, increasing reflection among both preceptors and students, and using failure as a catalyst for learning [14]. However, little research has been able to use these previous findings and suggestions to create a deeply meaningful intervention.

To overcome the limitations described in the literature and build on an educational alliance and coaching framework, a relational feedback approach can be utilized. *Relational feedback* is a term rooted in relational pedagogy—a teaching philosophy that aims to create a trusting and caring relationship that supports students throughout their educational journey [15]. Using this bidirectional caring relationship as a basis to give and receive feedback may improve the feedback process and forms the basis for this intervention.

### Research Purpose and Questions

To date, few studies have examined a relational feedback intervention for preceptors and students that focuses on their shared responsibilities and skills in the feedback process. This type of intervention brings medical students (feedback receivers) and preceptors (feedback givers) into the same room, with participants learning about feedback giving and receiving skills with and from each other through collaborative educational activities. They will be provided with educational tools for giving and receiving feedback within a coaching-like relationship wherein the feedback process is a 2-way conversation.

In this study, the following subquestions and objectives will be explored. First, what is the impact of a relational feedback intervention on the quality of feedback between preceptors and students during a longitudinal integrated clerkship (LIC)? We will explore up to 6 key elements of quality feedback conversations that were previously identified and validated by the Center for Medical Simulation [16]—(i) the establishment of an engaging learning environment, (ii) the maintenance of an engaging learning environment, (iii) feedback conversations organized in a structured way, (iv) the provocation of an engaging discussion, (v) the identification and exploration of performance gaps, and (vi) assistance in achieving or sustaining good future performance.

Second, what are the relational attributes that influence feedback incorporation and engagement with learning? We will investigate this from both learners’ and preceptors’ points of view. Through the presurveys, postsurveys, and follow-up surveys, we will examine the extent to which preceptors in LIC communities see positive attributes (eg, enthusiasm, openness, and collaboration) in their relationships with and supervision of their learners.

Third, in what ways does a relational feedback and professional development educational intervention influence the educational experiences of preceptors and students within the clinical settings of an LIC? As part of the intervention, we will explore this through facilitated discussion and developmental evaluation. We will also explore changes in both individual behaviors and community cultural patterns through presurveys, postsurveys, and follow-up surveys.

Fourth, what are the system-based factors that contribute to the impact of a relational feedback and professional development educational intervention? Again, we will explore this through both facilitated discussions during the relational feedback intervention and follow-ups with students and preceptors at the end of the LIC. This question and approach acknowledge that the wider context plays a crucial role in developing successful feedback relationships. They will allow us to understand the limits of a student-preceptor intervention and identify the enablers and barriers that intersect with knowledge and skills in the real-world context of clerkships in Northern Ontario.

We hypothesize that by creating awareness of the relationship context for both preceptors (feedback givers) and students (feedback receivers) and by providing tools for navigating that relationship, the intervention will be more successful. We also hypothesize that both student participants and preceptor participants will receive more effective feedback (quality) and additional feedback (quantity), thereby providing them with more opportunities to improve.

By answering these questions, we aim to improve medical students' educational and clinical experiences by further developing preceptors’ and students’ feedback skills and optimizing their relationships.

## Methods

### Context

The Northern Ontario School of Medicine (NOSM) was founded with a social accountability mandate to address physician shortages in Northern Ontario. The school aims to reach this goal by recruiting students who are interested in rural practice and giving these students positive rural educational experiences [17]. In its undergraduate curriculum, the NOSM uses a distributed model of learning, which is embedded with experiential learning experiences, wherein students undertake several placements in rural and remote communities [17]. In the third year, the core curriculum includes an 8-month LIC. During the clerkship, learners spend extended time in a community clinical setting where they have frequent and consistent interactions with their preceptors [18]. Within this educational context, it is of the utmost importance that students and preceptors develop relationships in which feedback can be given and received in a safe and effective manner.

### Sample

**Figure 1:**
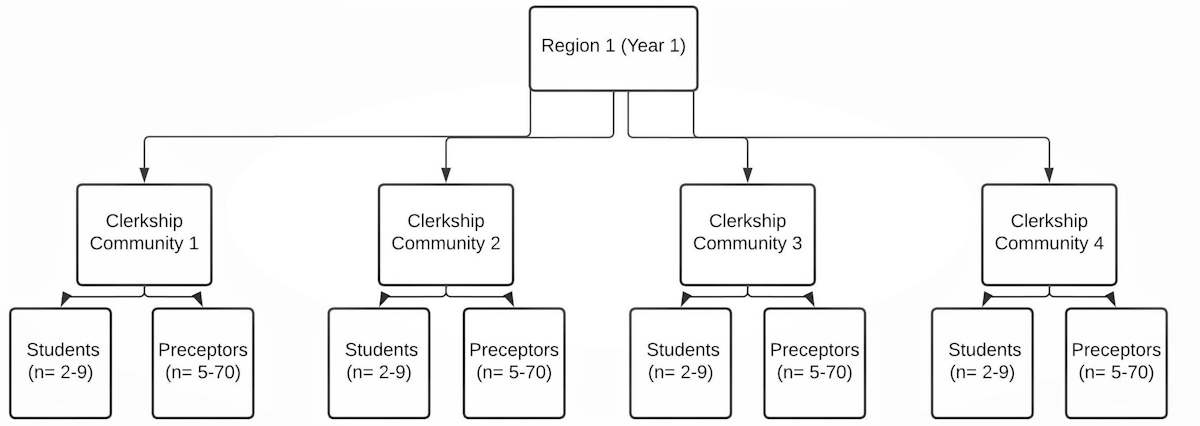
A visual representation of a sample year of interventions with clerkship communities and the range of the number of students and preceptors in each community.

The project was scheduled to last for 3 years (2019-2022) but has been extended to the end of 2023 due to the impact of COVID-19. Based on the geographic distribution of the clerkship communities, a regional approach will be utilized, and clerkship communities will be divided into 3 regions. Each year, 1 region, which includes 4 to 6 of the clerkship communities, will be visited until all 3 regions and 15 communities have participated. Communities range in size from 5000 to 76,000 people and include rural, semirural, and urban environments. There are between 2 and 9 medical students and between 5 and 70 preceptors in each clerkship community, as shown in Figure 1. JG will work with the site liaison clinician (academic lead) and site administrative coordinator to set up the educational intervention. The site administrative coordinator will send preceptors and students an invitation for participating in the research. Therefore, potential participants will be students and preceptors in each of the communities where the relational feedback intervention will be held. Since the study uses a pre- and posttest design, the participants who are interested in the study will form the intervention group. We will also invite other professionals who support teaching in the community, such as site educational administrators. Each research participant will be sent a letter of information and detailed consent forms before the project relational feedback intervention is performed. Participants will be notified about any potential risks and benefits and (for preceptors only) their responsibilities if they choose to participate by releasing their anonymized learner evaluations for analysis.

### Ethics Approval

This project has received approval from the Lakehead University Research Ethics Board (reference number: 8777) and Laurentian University Research Ethics Board (reference number: 6020466). To mitigate risk during COVID-19 outbreaks, sessions may be held over the internet via WebEx (Cisco Webex), depending on local risk and public health guidelines.

### Intervention

The intervention was designed by a group of community physicians, medical educators, and researchers at the NOSM, and to date, we have not found any comparable feedback professional development initiatives. Throughout this paper, the phrase *relational feedback intervention* is used to refer to the student-only, 1-hour orientation session; the 2.5-hour combined preceptor and student professional development session; and the 1-hour, student-only debrief/focus group.

Before the combined professional development session, students will be asked to participate in a 1-hour session that will provide an orientation on their role in receiving feedback and their participation in the combined session; they will discuss what makes feedback effective or difficult and how preceptors can make giving and receiving feedback more effective and safer for students.

Students and their preceptors will then participate in a 2.5-hour combined professional development session with the following learning objectives: (i) implement strategies for building preceptor-learner trust and rapport during feedback conversations within a clinical setting, (ii) provide feedback through coaching conversations to support the improvement of learners’ future performance, and (iii) use the Ask/Tell/Ask feedback framework to facilitate 2-way feedback conversations and assess the results. This will be accomplished through a 4-part outline.

For part 1, the concept of feedback and its impact on learners’ performance within a clinical setting will be introduced. Participants will describe successful examples of when they gave or received feedback during a clinical placement and identified factors that contributed to the success of the feedback. Participants will also describe difficult interactions in which they gave or received feedback during a clinical placement and factors that contributed to the difficulty. A facilitated discussion will be conducted to explore the conditions that make it safe for participants to both give and receive critical feedback on their performance.

For part 2, the Ask/Tell/Ask framework will be introduced. The Ask/Tell/Ask framework is a collaborative communication approach that allows a learner to explain their perceptions of their performance, receive feedback on their performance, and create a plan for improvement [19]. This framework will be introduced by using a 3-minute video that describes the cons of the feedback sandwich (“good/bad/good”); presents the purpose of feedback; provides an explanation of the Ask/Tell/Ask framework, which includes specific questions that can be asked (ie, what went well and what could have gone better in that patient encounter); and shows an example of the framework. The facilitators will use a video from the Virginia Apgar Academy of Medical Educators to spark discussions between preceptors and students [20].

For part 3, coaching techniques for linking feedback conversations with the development of opportunities for improving future performance will be discussed. Large groups will be divided into groups of 3 to 5 participants, who will work through 3 simulated/practice feedback examples. Each group will typically have 1 student and 2 to 3 preceptors. Each preceptor participant will be the preceptor, or observer, for one of the examples, while students will be the learners. Practice examples will provide opportunities to apply the Ask/Tell/Ask framework.

For part 4, following a large group debrief of 3 simulated/practice feedback examples, a discussion of strategies or next steps for supporting the development of preceptor-learner trust and rapport for future feedback conversations in a clinical setting will conclude the session.

The relational feedback intervention will be delivered by 2 members of the research team. EC is a professionally trained teacher with years of experience in facilitating workshops, an educational scholar, and an associate professor at the NOSM. JG is a clinician-scientist and the associate dean of Continuing Education and Professional Development, and one of the primary focuses of his research program is optimizing feedback conversations.

### Data Collection

**Figure 2:**
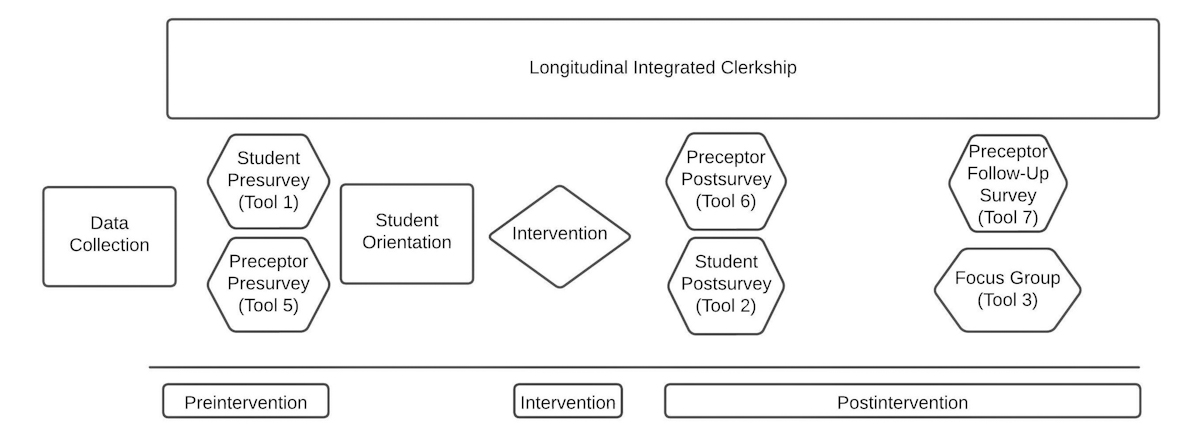
A visual representation of the data collection procedure.

To evaluate the relational feedback intervention, multiple tools were developed by the research team based on current literature in the field of medical education to answer the specified research questions. Standardized NOSM evaluations will also be used in the study. These tools are based on face validity and a data collection policy whereby information about the exact tools cannot be made available. The general timing and use of the data collection tools are shown in Figure 2.

### Research Tools

#### Student Precombined Professional Development Session Survey (Tool 1)

The student precombined professional development session survey includes a series of 4 open-ended questions on what makes preceptor feedback effective for improving performance, what aspects of receiving feedback are the most difficult for students, how preceptors can make it safe for students to receive critical feedback, and how preceptors can make it safe for students to provide feedback on their teaching or supervision.

#### Student Postcombined Professional Development Session Survey (Tool 2)

The student postcombined professional development session survey is a series of 7 open-ended questions regarding students’ perspectives on the combined professional development session and on attending it with preceptors, the strategies they learned to enhance both the giving and receiving of feedback, and how the NOSM can further assist with improving their skills for receiving and giving feedback.

#### Student Postcombined Professional Development Session Focus Group (Tool 3)

Students will participate in a debrief focus group wherein a semistructured guide will be used that asks questions on the lessons learned from the combined professional development session; how a relational approach supports teaching and learning, influences educational environments, and supports clinical practice; how the combined professional development session influenced competence and confidence in giving feedback; how the combined professional development session challenges previously held ideas; and whether students will be able to incorporate the material into their practice.

#### Student Program Evaluation Surveys (Tool 4)

During the 8-month LIC, students will complete an anonymous program evaluation survey 3 times. At the end of the clerkship, students will also be asked to complete an anonymous final program evaluation. These program surveys were not specifically designed for our study, but through a secondary analysis, useful information can be extracted to help us answer the project’s research questions. Both the end-of-year survey and program evaluation survey comprise a combination of open- and close-ended questions. The close-ended questions are typically yes-no questions or 4-point or 5-point Likert scale questions. Project-relevant questions from the program surveys include questions on how the clerkship experience can be enhanced (open ended), questions that ask students to comment on the usefulness of the feedback they have received (open ended), and questions on the learning environment (4-point Likert scale).

#### Preceptor Precombined Professional Development Session Survey (Tool 5)

The preceptor precombined professional development session survey asks a total of 4 open-ended questions on the successful provision of feedback, a difficult interaction in which preceptors provided feedback, what preceptors would like to learn about preceptor-learner feedback interactions, and what skills related to giving or receiving feedback are important to develop.

#### Preceptor Postcombined Professional Development Session Survey (Tool 6)

Like the student survey, the preceptors will be asked a series of 7 open-ended questions on general feedback about the combined professional development session and their thoughts on attending it with students. The questions ask about the strategies they learned to enhance both the giving and receiving of feedback and how the NOSM can further assist them in improving their skills for receiving and giving feedback.

#### Preceptor Follow-up Survey (Tool 7)

Approximately 6 to 8 weeks after the combined professional development session, preceptors will be sent a survey with 4 open-ended questions. The questions utilize previously gathered information and ask about the steps that were taken to make changes in giving feedback and whether these changes have led to any results. A series of probative questions about the last time preceptors gave feedback and further program development questions are also included in the survey.

#### Student Evaluations of Preceptors (Tool 8)

The research will also use secondary data from clinical faculty evaluations, which will be completed by NOSM learners. Preceptors will be asked to consent to the release of their completed evaluations, and only evaluations from consenting preceptors will be used. All NOSM undergraduate and postgraduate learners will be asked to complete clinical faculty evaluations on preceptors. Faculty evaluations include a combination of open- and close-ended Likert scale questions. The open-ended questions ask about areas of strengths and weaknesses, while close-ended questions ask about supervision, feedback, teamwork, and learning supportiveness. Information on students who complete the evaluations will not be recorded when the evaluations are submitted (ie, the evaluations will be anonymized at the time of submission).

### Analysis

The qualitative data from the surveys, interviews, focus groups, and preceptor evaluation forms will be transcribed verbatim, anonymized, and uploaded into ATLAS.ti (Scientific Software Development GmbH). To help protect anonymity and aid the analysis, each of the 4 to 6 communities will be treated as 1 cohort. Data will be coded by using grounded theory approaches and thematic coding. The grounded theory approaches will include open, axial, and selective coding, which will involve breaking data up into smaller sections, deeply analyzing these sections, developing codes, and drawing connections between codes [21]. The thematic coding process will follow the 6-step process described by Braun and Clark [22] (data familiarization, the generation of initial codes, the search for themes, the review of themes, the defining of themes, and the write-up). Depending on the specifics of the research questions, appropriate steps for ensuring quality will be included. These may include triangulation, the involvement of multiple researchers, audit trails, reflexivity, and accurate transcriptions [23]. Most analyses will focus on the qualitative data, but quantitative data will be analyzed by using descriptive statistics. Since the study involves more than 1 cohort, as well as data from preceptors and students, analyses will be performed within and across groups.

## Results

Participant recruitment began in January 2019. As of April 2021, a total of 29 students and 66 preceptors have completed the premeasures, postmeasures, and follow-up measures in 7 sites. We aim to finish the study in December 2023 and make the results available in 2024.

## Discussion

The relational feedback intervention will provide further knowledge on and promote growth in the conceptualization of feedback dialogues wherein learners and preceptors develop conversations and are equal participants in the feedback process. As both the students and the preceptors will be included in a combined professional development session where they learn with and from each other, there is the potential to amplify learning, enhance professional relationships, create a safer educational space for both students and preceptors, and improve feedback dialogues.

On the basis of the use of data from the pre– and post–professional development session surveys for both preceptors and students, we hypothesize that within the community clinical site, preceptors and students will learn new skills for giving and receiving feedback in a coaching relationship. Previous literature has noted problems with a lack of engagement from preceptors and difficulties with establishing relationships [11,12]. Having both the students and preceptors, along with other preceptors in the clerkship community, attend the combined session will increase its potential impact, as it will demonstrate the commitment to the feedback process in the clerkship community, help with creating coconstructed feedback guidelines, and build relationships through the educational activities.

On the basis of the postsession follow-up surveys, we hypothesize that in the medium term, preceptors will be able to incorporate feedback strategies within their clinical educational contexts, resulting in feedback that students are able to recognize, identify as useful, and effectively process. This finding will be interpreted by using social learning theory. Social learning looks at learning that takes place in a social context and how people learn from each other, and it has been used in medical education [24-26]. Preceptors in each clinical site function as a community of practice. As all preceptors will be invited to and given study credits for their attendance in the combined 2.5-hour professional development session, the lessons learned from the innovative intervention will diffuse throughout each community of practice, in part due to the participants learning from one another.

In the long term, there will be increased competence in relational feedback skills that students will apply in future clinical placements and preceptors will incorporate into their supervision of future learners. At the institutional level, the design of the study (ie, the inclusion of all community clinical sites) will help with creating a safer place for giving and receiving feedback. The results of the study will be of interest to the medical education community and other health care professionals, as feedback is an integral part of career training. Implementing the relational feedback intervention will allow for improvements in learners’ and preceptors’ relationships, which will enhance their learning environments.

Our study will have some potential limitations. First, the study will use secondary data from student evaluations of preceptors. As interventions will occur at different times in the year, there is potential for recency bias in the student evaluations. Students that have the intervention closer to a teaching evaluation might report more favorable outcomes related to feedback. Another limitation of the study and other educational studies is the system's noted complexities. There is a chance that we may miss some of the system-level variables that influence the feedback relationship.

At the conclusion of the project, the research team will be uniquely positioned to disseminate the results of the project within the institution and beyond. The team includes practicing health professionals who can disseminate the findings to other health professionals, individuals who work in continuing professional development and can share the results with their administrations, and academics who can help with presenting the results at academic conferences and publishing the results in peer-reviewed journals. The findings will also be presented in 1-page infographics that will be distributed over social media.

## Abbreviations

LIC: longitudinal integrated clerkship
NOSM: Northern Ontario School of Medicine
